# Utah Homœopathic Medical Association

**Published:** 1892-07

**Authors:** M. J. Green


### UTAH HOMŒOPATHIC MEDICAL ASSOCIATION.

### Proceedings of the First Annual Session.

The Utah Homœopathic Medical Association convened in First Annual Session May 3d, 1892, in the parlors of the Metropolitan Hotel, Salt Lake City, Utah.

There were present the following members: Drs. J. M. Dart, J. Beattie, C. Q. Douglas, H. H. Crippen, D. A. Sykes, C. C. Shinnick, G. V. Parmelee, E. D. Woodruff, J. C. Hanchett, C. L. Crandall, J. T. White, all of Salt Lake City; Drs. Ried and Graham, of Ogden; Dr. Brant, of Eureka. The following visitors were in attendance: Dr. Mary J. Green, Salt Lake City; Dr. C. W. Clark, of Eureka; Dr. Walker, of Nebraska, and Dr. Ireland, of Ohio.

The Association was called to order at 10.30 A. M., by the President, Dr. J. M. Dart. The report of the Executive Committee was presented and accepted.

The annual address was received with great satisfaction, and referred to a committee of three, consisting of Drs. Crippen, Shinnick, and Crandall.

The following from the Bureau of Materia Medica and Therapeutics were read and discussed:

"The Two Materia Medicas," by the Chairman, J. C. Hanchett, M. D., Salt Lake City; "The Therapeutics of Acute Conjunctivitis," H. H. Crippen, M. D., Salt Lake City; "Electricity in Medical Practice," W. F. Howe, M. D., Evanston, Wyoming; "Some Peculiar and Persistent Symptoms with Clinical Notes," H. W. Brant, M. D., Eureka, Utah; "Positive Therapeutics and Homœopathy," J. Beattie, M. D., Salt Lake City.

In the course of the discussion Dr. Crippen moved that the privileges of the Association be extended to the visiting physicians. This was adopted, and the Chairman asked Drs. Green, Clark, Walker, and Ireland to assist in the consideration of the subjects presented.

The following officers were elected for the ensuing year, by acclamation : J. Beattie, M. D., President; C. L. Crandall, M. D., Vice-President; J. T. White, M. D., Secretary; H. H. Crippen, M. D., Corresponding Secretary; J. C. Hanchett, M. D., Treasurer. Board of Censors—Drs. Graham, C. L. Douglas, and C. L. Crandall.

The Board of Censors reported with recommendation for election to membership the following: M. J. Green, M. D., Salt Lake City; C. W. Clark, M. D., Eureka, and were elected members by an unanimous vote.

The following from the Bureau of Clinical Medicine and Surgery were read and discussed:

"A Clinical Case in Surgery," by the Chairman, G. V. Parmalee, Salt Lake City; "Dressing and Treatment of Contused and Lacerated Wounds," H. W. Brant, M. D., Eureka; "Clinical Cases in Surgery," E. B. Graham, M. D., Ogden; "Abscesses; Metastatic and General," D. A. Sykes, M. D., Salt Lake City; "Orificial Surgery,," C. C. Shinnick, M. D., Salt Lake City; "Inflammation in the Region of the Cæcum," C. L. Crandall, M. D., Salt Lake City.

Through the courtesy of the Chairman, the President-elect appointed the following:

J. M. Dart, M. D., Chairman of the Bureau of Materia Medica and Therapeutics.

E. L. Douglas, M. D., Chairman of the Bureau of Clinical Medicine and Surgery.

D. A. Sykes, M. D., Chairman of the Bureau of Obstetrics, Gynæcology and Pædology.

Committee on Legislation—Drs. Crandall, Shinnick, Ried, Douglas, and Graham.

Committee on Organization and Statistics—Drs. Parmelee, Crippen, Hanchett, White, and Woodruff.

The Committee on President's Address submitted the following:

"Our President has given us a very enjoyable relation of the early struggles of Homœopathy in Utah. A history which will, we trust, incite us to united efforts toward placing this Association in a position to demand legislative recognition in the distribution of future favors to be bestowed upon the profession." Accepted.

Salt Lake City was selected as the place in which the next annual session will be held.

The following were read and discussed in the Bureau of Gynæcology, Obstetrics, and Pædology :

" Practical Considerations of late Gynæcological Topics," by the Chairman, C. L. Crandall, M. D., Salt Lake City ; " The Relation of Eye-Strain to Some of the Nervous Reflexes of Childhood," H. H. Crippen, M. D., Salt Lake City ; " Cervical Endometritis," C. I. Douglas, M. D., Salt Lake City ; " Practical Points in Infant Feeding."

Minutes of Organization meeting read and accepted.

On adoption of motion, it was decided to accept the report, reserving to the next meeting the settlement of the question of forming a Clinical society.

After attending to other matters of unfinished business, the Association adjourned to meet on the second Tuesday in June.

The Association was royally entertained in the evening by Dr. J. M. Dart, at his residence, 553 E. Second South Street.

All did justice to the delicious repast, after which we were favored with some rare music. During the festivities of the evening, a telegram was received by Dr. J. M. Dart, from the Homœopathic Medical Association of New Jersey, then in session at Trenton, extending a greeting to the Utah Medical Association, and welcoming her as her youngest sister.

At a late hour the company dispersed, feeling that they had spent a profitable day and a most enjoyable evening, and that Utah would yet win laurels for Homœopathy.

MRS. M. J. GREEN, M. D.

